# miR-181d-5p Protects against Retinal Ganglion Cell Death after Blunt Ocular Injury by Regulating NFIA-Medicated Astrocyte Development

**DOI:** 10.1155/2022/5400592

**Published:** 2022-10-08

**Authors:** Jinghua Li, Junyi Liu, Yuanping Zhang, Xu Zha, Hong Zhang, Yongying Tang, Xueying Zhao

**Affiliations:** Ophthalmology Department, The Second Affiliated Hospital of Kunming Medical University, Kunming, Yunnan 65000, China

## Abstract

**Background:**

Traumatic optic neuropathy (TON) refers to damage to the optic nerve resulting from direct and indirect trauma to the head and face. One of the important pathological processes in TON is the death of retinal ganglion cells (RGCs), but the cause of RGCs death remains unclear. We aimed to explore the mechanisms of RGCs death in an experimental TON model.

**Methods:**

Optic nerve crush injury was induced in ten New Zealand white rabbits. On the 1st, 3rd, 7th, 14th, and 28th days after the operation, the retinal tissues of the rabbits were observed pathologically by hematoxylin-eosin staining. The expression of POU-homeodomain transcription factor Brn3a and glial fibrillary acidic protein (GFAP) was measured by immunofluorescence to evaluate the number of RGCs and astrocytes, respectively. miRNA expression and protein levels were assessed by RT-qPCR and western blot methods, respectively. Finally, the malondialdehyde content, superoxide dismutase activity, and proinflammatory factor levels were measured by ELISA. Western blot and dual-luciferase reporter assays were used to elucidate the relationship between miR-181d-5p and nuclear factor I-A (NFIA).

**Results:**

Blunt ocular trauma increased oxidative stress and apoptosis and reduced ganglion cell layer (GCL) density. The expression of miR-181d-5p was decreased in retinal tissues, and its overexpression relieved RGCs death, astrocyte development, oxidative stress, and inflammation of the retina, which were reversed by NFIA overexpression.

**Conclusion:**

miR-181d-5p can protect against the deterioration of TON by inhibiting RGCs death, astrocyte development, oxidative stress, and inflammation by targeting NFIA. This study provides new insight into early medical intervention in patients with TON.

## 1. Introduction

Traumatic optic neuropathy (TON) refers to optic nerve damage that results from direct or indirect trauma to the head and face; this condition is rare but can cause severe and irreversible vision loss [1]. TON treatment is mainly divided into three methods: high-dose steroid therapy, surgical decompression, and combination steroid and optic nerve decompression therapy [1, 2]. Unfortunately, the reliance on these methods has been controversial [3, 4]. On the one hand, although complications of steroid therapy are rare, there is no obvious evidence that steroid therapy has benefit in improving vision in patients with TON [2]. On the other hand, surgical treatment of the optic nerve carries a clear risk of complications, such as postoperative cerebrospinal fluid leakage and meningitis, and there is no evidence that surgery produces any additional benefit [5]. Therefore, it is crucial to discover a new and effective therapeutic strategy for TON.

Retinal ganglion cells (RGCs) axons and supporting cells make up the optic nerve (ON) cells [6]. Because they are central neurons, RGCs lack endogenous regenerative capacity. Therefore, RGCs undergoing apoptosis cannot be replaced, and damaged ON cells cannot be regenerated, leading to irreversible blindness [7]. Trauma leads to a series of pathological events, including inflammation [8] and oxidative stress [9]. These risk factors are closely related to the apoptosis of RGCs. Research has found that RGCs death is associated with TON, and resveratrol treatment can delay the loss of RGCs and the loss of pupillary light response after optic nerve compression [8], suggesting that a protective strategy for RGCs may be a promising next-generation therapy. Most forms of neurological disease are associated with reactive astrocytes, ranging from acute injury to degeneration [10]. Impairment of axonal regeneration and functional recovery results from damage to the central nervous system, enabling the transformation of naive astrocytes into reactive astrocytes and ultimately into scar-forming astrocytes [11]. Interestingly, a recent study found that the injured ON promotes astrocyte accumulation, glial scar formation, and RGCs death in the retinal layer [12]. In addition, astrocytes are emerging as central regulators of retinal ON inflammatory responses because they also have strong proinflammatory potential [13]. However, how trauma promotes astrocyte accumulation remains largely unknown. It is important to investigate the influence of astrocyte aggregation and the inflammatory response on TON and its underlying mechanisms.

As a transcription factor, NFIA not only regulates astrocyte development but also affects inflammation [14] and oxidative stress [15]. These risk factors are strongly associated with the pathogenesis of TON [8, 9]. Considering the critical role of inflammation and oxidative stress in causing RGCs death, NFIA may regulate these processes and play a role in TON. In addition, it has been reported that the NFIA protein is highly expressed in reactive astrocytes during human neurological injury, such as multiple sclerosis, hypoxic-ischemic encephalopathy, and spinal cord [16–18], but the expression of NFIA in TON has not been reported. In particular, NFIA localizes the nucleus to the inner nuclear layer and the nerve fiber layer, thereby regulating retinal development [19], suggesting that NFIA is important in the development of the retina. However, whether NFIA regulates astrocyte accumulation in TON remains undefined.

In this study, we found that NFIA is upregulated in the retina of TON. miR-181d-5p is an upstream target of NFIA. miR-181d-5p inhibited RGCs loss and astrocyte development by downregulating NFIA. This study revealed a new molecular mechanism of RGCs death during the TON process and provided a potential therapeutic target for the development of new treatment methods for TON.

## 2. Materials and Methods

### 2.1. Animal Model and Treatment

New Zealand rabbits (clean grade) were obtained from the Experimental Animal Center of Southern Medical University (Guangzhou, China), and all rabbits were housed in a light-dark (12 : 12) cycle temperature-controlled barrier facility with enough food and water at a temperature of 22 °C and a humidity of 50%. All experiments and their protocols involved in this study were approved by the Institutional Committee for Animal Care and Utilization of Kunming Medical University (protocol reference No. kmmu20211586). As described previously [20], rabbits were anesthetized with 3% phenobarbital, and the neurobone canal (or ring) surrounding the optic nerve was isolated through an operating microscope. After inserting the Yasargil aneurysm clip (65742) into the bony canal (or bony ring) to clamp the nerve for 30 seconds, the clip was removed. In this study, rabbits were divided into the model group, sham group, or miR-181 (miR-181d-5p mimic) group and were treated for four weeks as follows: model rabbits receiving agomiR-181d-5p treatment as described previously (5 nmol agomiR-181d-5p applied 4 times daily) [21] and (4) miR-181+oe-NFIA (NFIA overexpression lentiviral vector) group: model rabbits receiving agomiR-181d-5p and oe-NFIA (Guangzhou RiboBio Biotechnology Co., Ltd., Guangzhou, China). All experimental rabbits were euthanized on days 1, 3, 7, 14, or 28 of treatment and their tissues were used for further analysis.

### 2.2. Hematoxylin-Eosin (H&E) Staining

The rabbit eyeballs were removed and fixed with 4% paraformaldehyde for 20 min, and 5 *μ*m thick tissue pieces were stained with H&E at 25 °C for 10 min. A Zeiss fluorescence microscope (Axio imager microscope) with identical acquisition settings was used to observe H&E staining (magnification 50x). Three sections were taken from each eye, and the data were analyzed by GCL cell density in each visual field.

### 2.3. Immunofluorescence

The rabbit eyeballs were fixed with FAS solution at room temperature for 24 h and sectioned into paraffin-embedded sections with a thickness of 5 *μ*m. The sections were dewaxed, heated at high pressure for antigen repair, sealed with goat serum at room temperature for 1 h, and incubated with primary antibodies against glial fibrillary acidic protein (GFAP) (dilution ratio, 1 : 1000; No. ab7260; Abcam, UK) and Brn3a (dilution ratio, 1 : 1000; No. ab245230; Abcam) at 4 °C overnight. After the addition of Alexa Fluor-conjugated secondary antibody, the cells were incubated at 25 °C for 1 h. Subsequently, cell nuclei were stained with 0.1% DAPI for 5 min at 25 °C. Immunofluorescence staining was visualized at 50x magnification using a Zeiss fluorescence microscope. Three sections were taken from each eye, and the data were analyzed by ganglion cell layer (GCL) cell density in each visual field.

### 2.4. Determination of Malondialdehyde (MDA) Content and Superoxide Dismutase (SOD) Activity

Retinal tissue from rabbits was harvested, and the MDA content and SOD activity in retinal tissue were determined using the corresponding ELISA kits (Sangon Biotech, Shanghai) following the manufacturer's instructions.

### 2.5. Western Blot Assay

The total proteins from retinal tissue were extracted utilizing RIPA lysis buffer (Sangon Biotech, Shanghai) and using a BCA assay (Sangon Biotech, Shanghai) to determine the total protein content. A 10% SDS-PAGE gel was used to separate the extracted total proteins, which were then transferred to polyvinylidene fluoride membranes by a constant current flow at 200 mA. Subsequently, PVDF membranes were incubated with Bcl-2 (1 : 500; No. ab196495; Abcam), Bax (1 : 1000; ab32503; Abcam), NFIA (1 : 1000; ab228897; Abcam), and GAPDH (1 : 5000; ab8245; Abcam) antibodies for 12 h at 4 °C after blocking with 5% skim milk. The PVDF membranes were washed with TBS buffer and incubated with corresponding secondary antibodies (Abcam) at 25 °C for 1 h. Immunoblots were visualized using chemiluminescent reagents (Yeasen, Shanghai, China), and grayscale analysis was performed by the ImageJ software.

### 2.6. RNA Extraction and RT-qPCR Assay

RNA was isolated from retinal tissue and RGCs using a Total RNA Extractor (Sangon Biotech) and a first-strand cDNA synthesis kit (Vazyme, Nanjing, China) to reverse transcribe it into cDNA. Subsequently, RT-qPCR was performed using a universal high-specificity, dye-based, quantitative PCR detection kit (Vazyme, Nanjing, China) in an ABI 7300 sequence detection system (Applied Biosystems) with thermal cycling conditions of 94 °C for 5 min, 40 cycles of denaturation at 94 °C for 15 s, and annealing at TM value (60 °C) for 30 s. The U6 gene was selected as the reference gene, and the relative expression of the target gene was calculated by the 2^-*∆∆*Ct^ method [22].

### 2.7. Dual-Luciferase Reporter Gene Assay

In this study, dual-luciferase reporter vectors containing wild-type (WT) and mutant-type (MUT) binding sites for NFIA sequences were constructed by a rapid cloning kit (Vazyme, Nanjing, China) and named WT-NFIA and MUT-NFIA, respectively. Subsequently, WT-NFIA and MUT-NFIA vectors were transfected into 293T cells (Chinese Academy of Sciences Culture Collection) with miR-181d-5p mimic and negative controls. After transfection for 48 h, the dual-luciferase reporter assay (Promega) was used to detect luciferase activity.

### 2.8. ELISA

In this study, following the instructions of the ELISA kit (Abcam), the retinal tissues of rabbits were collected after treatment, and the content of inflammatory cytokines, including TNF-*α*, IL-1, IL-1*β*, and IL-6, was measured at the corresponding wavelength (450 nm) optical density using a microplate spectrophotometer (BioTek, USA).

### 2.9. Statistical Analysis

The GraphPad Prism 8 software was used to analyze and prepare graphs of the experimental data. In this study, all data are shown as the mean ± standard deviation (SD). Data from two groups and multiple groups were analyzed by unpaired Student's *t*-test and one-way analysis of variance followed by Tukey's post hoc test, respectively. The *P* value for statistical significance was 0.05.

## 3. Results

### 3.1. Trauma Increases Retinal Oxidative Stress and RGCs Death

**Figure 1:**
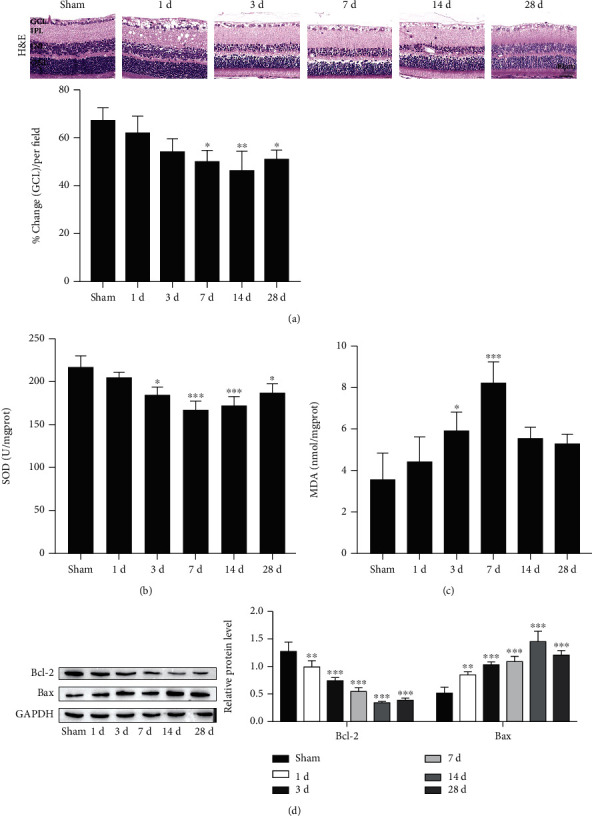
Trauma increases retinal oxidative stress and RGCs death. (a) Representative images of retinal tissues stained with hematoxylin and eosin (H&E; scale bar = 40 *μ*m). (b) Superoxide dismutase (SOD) activity. (c) Malondialdehyde (MDA) content. (d) Western blot detection of Bcl-2 and Bax proteins. ^∗^*P* < 0.05, ^∗∗^*P* < 0.01, and ^∗∗∗^*P* < 0.001 vs. the sham group.

To understand and investigate the potential impact of trauma on retinal laminar structure, retinal sections were histologically assessed by H&E staining in this study. As shown in Figure 1(a), GCL density gradually decreased with time after trauma, and the lowest point was measured at 14 d, which showed significant changes at 7 d, 14 d, and 28 d. The activity of SOD, an antioxidative enzyme, gradually decreased with time after trauma and reached the lowest point at 7 d (Figure 1(b)), while the content of MDA, an oxidative stress marker, gradually increased with time and reached the highest point at 7 d (Figure 1(c)). We further investigated the apoptotic effect of traumatic conditions in the retina. Western blotting was used to measure the expression of Bcl-2 and Bax. As represented in Figure 1(d), Bcl-2 gradually decreased with time after trauma and reached the lowest point at 14 d, while Bcl-2 gradually increased with time and reached the highest point at 14 d. As shown in Figures 1(a), 1(b), and 1(d), compared with the sham group, these data showed a large difference at 14 d. Thus, we chose 14 d after trauma for the following experiments. These results demonstrate that trauma increases retinal oxidative stress and RGCs death.

### 3.2. miR-181d-5p Is Expressed at Low Levels in Injured Retinal Tissues

**Figure 2:**
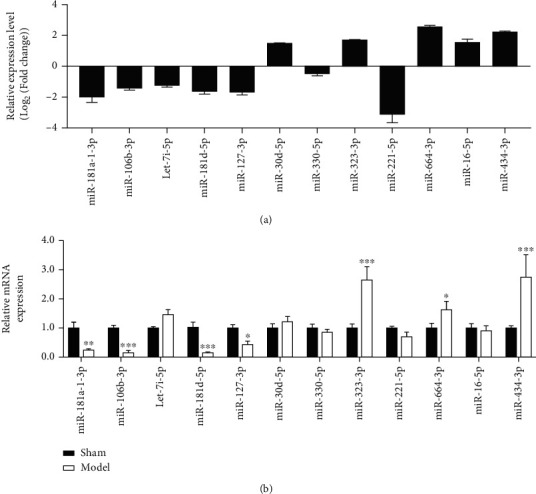
miR-181d-5p is expressed at low levels in injured retinal tissues and RGCs. (a) The RNA-seq data between normal RGCs and injured RGCs (7 days postoptic nerve crush) and selected the top 12 differentially expressed miRNAs. (b) Expression of 12 miRNAs in traumatic retinal tissue by RT-qPCR detection. ^∗^*P* < 0.05, ^∗∗^*P* < 0.01, and ^∗∗∗^*P* < 0.001 vs. the sham group.

MicroRNAs (miRNAs) can be involved in the pathophysiologic process of many diseases by binding to target mRNAs [23]. A recent study identified that ocular hypertension and TON induce significant changes in RGCs miRNAs [24]. To explore a functional miRNA in TON, we queried the RNA-seq data between normal RGCs and injured RGCs (seven days postoptic nerve crush) and selected the top 12 differentially expressed miRNAs; the expression of these 12 miRNAs was analyzed by RT-qPCR. Among these abnormally expressed miRNAs, we were concerned with miR-181d-5p, which is one of the lowest expressed miRNAs in injured RGCs (Figure 2(a)) and injured retinal tissues (Figure 2(b)). These data indicate that miR-181d-5p was highly expressed in traumatic retinal tissues and RGCs.

### 3.3. miR-181d-5p Relieves RGCs Death, Astrocyte Development, Oxidative Stress, and Inflammation of the Retina

**Figure 3:**
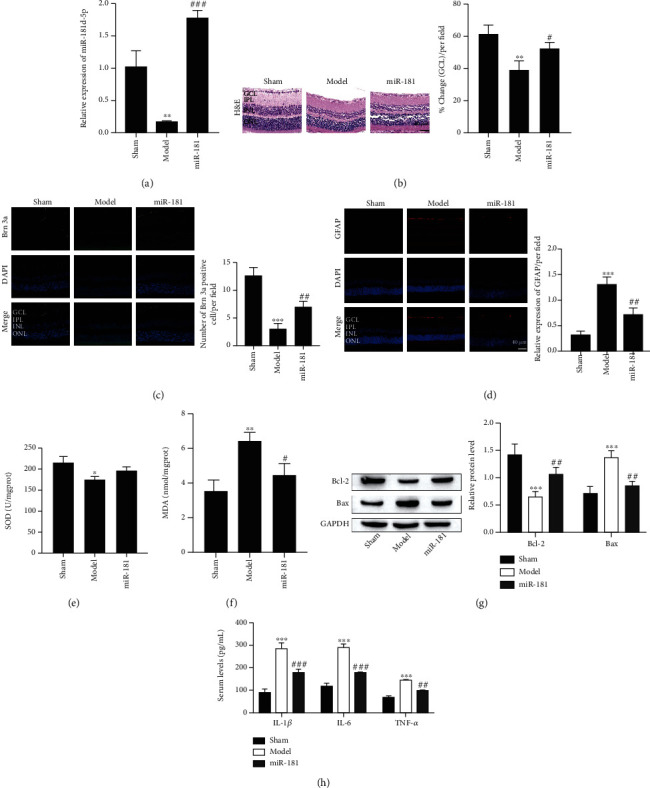
miR-181d-5p relieves RGCs death, astrocyte development, oxidative stress, and inflammation of the retina. (a) Expression of miR-181d-5p by RT-qPCR detection. (b) Representative images of retinal tissues stained by H&E (scale bar = 40 *μ*m). (c) Representative images of POU-homeodomain transcription factor Brn3a staining (scale bar = 40 *μ*m). (d) GFAP staining (scale bar = 40 *μ*m). (e) SOD activity. (f) MDA content. (g) Western blot assay of Bcl-2 and Bax protein. (h) Proinflammatory factor levels were measured by ELISA. ^∗^*P* < 0.05, ^∗∗^*P* < 0.01, and ^∗∗∗^*P* < 0.001 vs. the sham group; ^#^*P* < 0.05, ^##^*P* < 0.01, and ^###^*P* < 0.001 vs. the model group.

We next overexpressed miR-181d-5p in rabbit eyeballs (Figure 3(a)). miR-181d-5p overexpression dramatically increased the GCL density of the retina compared with that of the model group (Figure 3(b)). RGCs death and astrocyte development are important indicators of TON [25, 26]. Immunofluorescence assays showed that the expression of POU-homeodomain transcription factor Brn3a, an RGCs marker, was reduced in the injured retinal tissues but was increased by miR-181d-5p overexpression (Figure 3(c)). In contrast, as an astrocyte marker, GFAP was elevated in the traumatic retinal tissues, and the increase was rescued by miR-181d-5p overexpression (Figure 3(d)). In addition, miR-181d-5p overexpression remarkably enhanced SOD activity and reduced MDA content (Figures 3(e) and 3(f), respectively). The expression of Bcl-2 was increased, while the expression of Bax was decreased under the overexpression of miR-181d-5p (Figure 3(g)). We further tested the content of TNF-*α*, IL-1*β*, and IL-6 in the retinal tissues by ELISA. We noticed that their levels were elevated after trauma but this elevation was reduced by upregulating miR-181d-5p (Figure 3(h)). It was discovered that miR-181d-5p rescues RGCs death, oxidative stress, astrocyte development, and inflammation in retinal cells.

### 3.4. Effect of miR-181d-5p on NFIA

**Figure 4:**
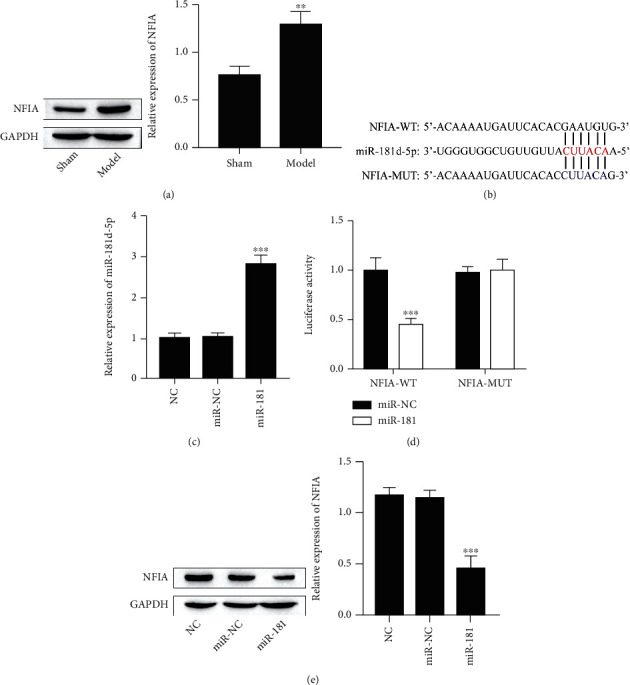
miR-181d-5p directly targeted the NFIA gene. (a) Representative western blot of nuclear factor I-A (NFIA). ^∗∗^*P* < 0.01 vs. the sham group. (b) The binding sites of miR-181d-5p in the NFIA sequence by starBase prediction. (c) Expression of miR-181d-5p by RT-qPCR detection. ^∗∗∗^*P* < 0.001. (d) The interaction between the NFIA 3′UTR and miR-181d-5p was analyzed by a dual-luciferase reporter gene. 293T cells were cotransfected with miR-181d-5p mimic and WT-NFIA or miR-181d-5p mimic and MUT-NFIA. ^∗∗∗^*P* < 0.001. (e) Representative western blot of NFIA. ^∗∗∗^*P* < 0.001.

NFIA was upregulated in the injured retinal tissues (Figure 4(a)). Identifying the targets of miR-181d-5p is a very important part of this mechanism. In this study, starBase v2.0 software (http://starbase.sysu.edu.cn/targetSite.php) was used to predict miR-181d-5p Targeted binding site with NFIA (Figure 4(b)) [27]. Meanwhile, to confirm the interaction function between miR-181d-5p and NFIA, WT or MUT-3′UTR of NFIA was cloned into the luciferase reporter vector. We next overexpressed miR-181d-5p in 293T cells (Figure 4(c)), other than the finding that the luciferase activity of the WT-NFIA reporter vector was significantly restrained by the overexpression of miR-181d-5p (Figure 4(d)). In addition, the expression of NFIA was significantly reduced in 293T cells transfected with miR-181d-5p mimic (Figure 4(e)). These data indicate that miR-181d-5p directly targets the NFIA.

### 3.5. miR-181d-5p Relieves RGCs Death, Astrocyte Development, Oxidative Stress, and Inflammation by Downregulating NFIA

**Figure 5:**
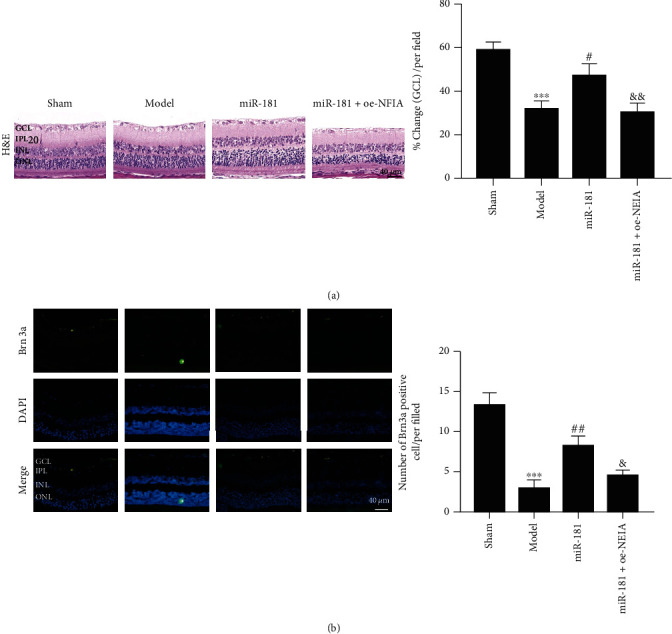
miR-181d-5p relieves RGCs death by targeting NFIA. (a) Representative images of retinal tissues stained with H&E (scale bar = 40 *μ*m). (b) Representative images of POU-homeodomain transcription factor Brn3a staining (scale bar = 40 *μ*m). ^∗∗∗^*P* < 0.001; ^#^*P* < 0.05, ^##^*P* < 0.01; ^&^*P* < 0.05, ^&&^*P* < 0.01.

**Figure 6:**
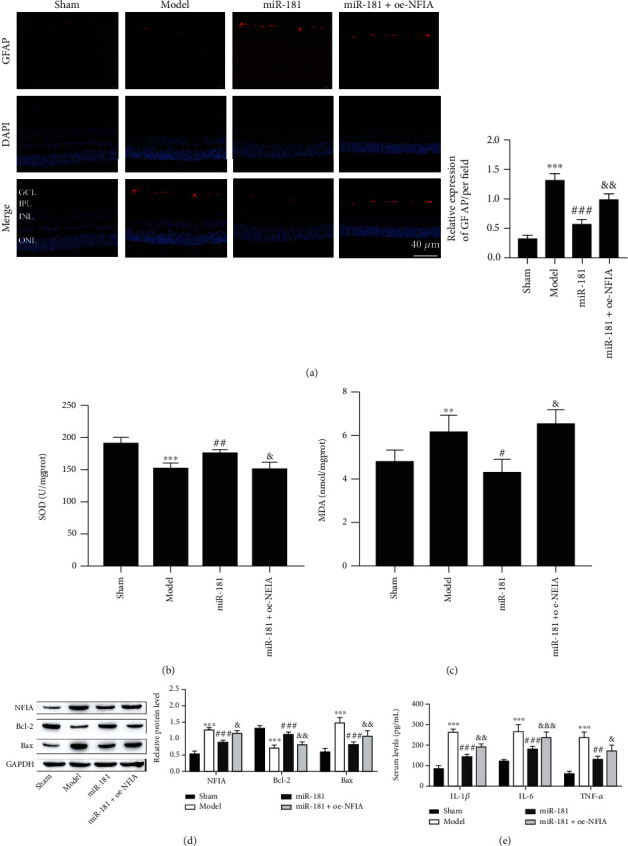
miR-181d-5p relieves astrocyte development, oxidative stress, and inflammation by targeting NFIA. (a) GFAP staining (scale bar = 40 *μ*m). (b) SOD activity. (B) MDA content. (d) Western blot of Bcl-2 and Bax protein. (e) Proinflammatory factor levels were measured by ELISA. ^∗∗^*P* < 0.01 and ^∗∗∗^*P* < 0.001 vs. the sham group; ^#^*P* < 0.05, ^##^*P* < 0.01, and ^###^*P* < 0.001 vs. the model group; ^&^*P* < 0.05, ^&&^*P* < 0.01, and ^&&&^*P* < 0.001 vs. the miR-181 group.

Next, to determine the effects of the miR-181d-5p/NFIA axis on RGCs death, astrocyte development, oxidative stress, and inflammation of the retina, we used miR-181d-5p mimics and an NFIA overexpression vector to change miR-181d-5p and NFIA expression. As shown in Figure 5(a), trauma lowered GCL density, which was reversed with overexpression of miR-181d-5p, but GCL density was ultimately repressed by overexpression of NFIA. Immunofluorescence analysis showed that Brn3a expression was significantly inhibited by induced trauma but enhanced after miR-181d-5p overexpression, which was terminally repressed by upregulating NFIA (Figure 5(b)). In contrast, GFAP protein levels were elevated after induced trauma but reduced with miR-181d-5p overexpression, which was terminally boosted by NFIA overexpression (Figure 6(a)). Overexpression of miR-181d-5p alleviated the trauma-induced SOD activity reduction and MDA content increase, and NFIA overexpression reversed the effect of miR-181d-5p overexpression (Figures 6(b) and 6(c)). The expression of NFIA was elevated after induced trauma but reduced with miR-181d-5p overexpression, which was terminally boosted by NFIA overexpression (Figure 6(d)). The expression of Bax showed a similar trend as NFIA expression, but the expression of Bcl-2 showed the opposite trend (Figure 6(d)). Finally, the levels of TNF-*α*, IL-1*β*, and IL-6 were elevated after induced trauma but reduced with miR-181d-5p overexpression, which was terminally boosted by NFIA overexpression (Figure 6(e)). Taken together, our data show that miR-181d-5p relieves RGCs death, astrocyte development, oxidative stress, and inflammation by downregulating NFIA.

## 4. Discussion

Trauma to the head and face can directly or indirectly cause damage to TON cells [1]. The major cellular component of the retina is RGCs, the loss of which can lead to retinopathy, including photoreceptor degeneration, diabetic retinopathy, glaucoma, and TON [28–30]. In our study, we observed RGCs death, astrocyte development, oxidative stress, and inflammation in the retinas of the TON animal model. A recent study identified that ocular hypertension and TON induce significant changes in RGCs miRNAs [24]. Through RT-qPCR analysis, we identified the top 12 differentially expressed miRNAs in retinas and RGCs. Among these abnormally expressed miRNAs, we identified miR-181d-5p to play a critical role in RGCs death in TON. According to previous reports, miRNAs are involved in gene regulation and other cellular processes and play a wide range of roles [23], including RGCs death [31]. Here, we found that miR-181d-5p targets the NFIA gene. Notably, accumulating evidence shows that NFIA levels regulate astrocyte development, oxidative stress, and inflammation [14, 15, 17]. Overexpression of miR-181d-5p relieves RGCs death, which may be related to astrocyte development, oxidative stress, and inflammation, suggesting that miR-181d-5p and NFIA are critical for preventing RGCs death in the retina after induced trauma.

In the process of retinal degeneration, cellular oxidative stress plays an indispensable role, including TBI and TON [9]. Our results indicate that the overexpression of miR-181d-5p alleviated the trauma-induced SOD activity reduction and MDA content increase in this study. Previous studies have shown that with the increase in oxidative stress in the mouse retina, RGCs death was decreased by TON and inhibition of oxidative stress [8, 32]. We utilized the expression of Brn3a measured by immunofluorescence to evaluate RGCs death, and Brn3a was significantly decreased in the model group, while miR-181d-5p increased this value, indicating that miR-181d-5p has the ability to prevent RGCs loss. Notably, as a rheostat, Bcl-2/Bax can regulate cellular antioxidant pathways and death [33]. We observed that miR-181d-5p promoted the expression of Bcl-2 while decreasing the expression of Bax. Our study supports this view and suggests that miR-181d-5p may inhibit oxidative stress-linked RGCs death, which is beneficial for TON prevention.

Astrocytes have extensive proinflammatory capabilities and are regulators of inflammatory responses in the CNS [13]. Reactive astrocyte accumulation has been hypothesized to underlie RGCs apoptotic processes after TON [12]. In addition, studies have shown that excessive activation of astrocytes is detrimental to the repair of retinal ganglion cells after optic nerve injury [34]. Inhibition of neuroinflammatory reactive astrocyte formation also significantly reduced the death of RGCs in mouse models of glaucoma [35]. In our study, significant astrocyte development in the retina of TON animal models was observed and miR-181d-5p effectively reduced the number of astrocytes. Moreover, the levels of TNF-*α*, IL-1*β*, and IL-6 were elevated after induced trauma but were reduced with miR-181d-5p overexpression. Furthermore, oxidative stress in astrocytes and neurons is triggered by astrocyte dysfunction which may lead to neurodegeneration [36]. Here, we found that the number of astrocytes was elevated in the retina of TON animal models, and that the oxidative stress response was also increased.

Interestingly, NFIA is a transcription factor that not only regulates astrocyte development but also affects inflammation [14] and oxidative stress [15]. NFIA in astrocytes has an endogenous prodifferentiation function [37], and NFIA is highly expressed in astrocytes responsive to human neural injury [17]. In addition, miRNA can play a role through targeted regulation of NFIA, for example, miR-424 can prevent astrocyte proliferation after cerebral ischemia/reperfusion in elderly mice by regulating NFIA [38]. In the present study, we determined that the expression of BFIA was upregulated in the retinal tissues of TON animal models. However, miR-181d-5p rescued the upregulated expression of NFIA. Furthermore, overexpression of NFIA reversed the effects of miR-181d-5p against RGCs death, astrocyte development, oxidative stress, and inflammation. These results indicated that miR-181d-5p protects against TON by downregulating NFIA. In this study, RGCs death and associated astrocyte activation were observed in traumatic optic neuropathy, which was consistent with previous studies, but this study proposed a new regulatory mechanism and for the first time explored the effect of mir-181d-5p/NFIA molecular axis on RGCs death.

In conclusion, miR-181d-5p can protect against the deterioration of TON by inhibiting RGCs death, astrocyte development, oxidative stress, and inflammation through downregulation of NFIA. These results provide new insights for early medical interventions in patients with TON.

## Data Availability

The datasets used and/or analyzed during the current study are available from the corresponding author upon reasonable request.
